# Antioxidant, Anti-Inflammatory and Antiproliferative Effects of *Osmanthus fragrans* (Thunb.) Lour. Flower Extracts

**DOI:** 10.3390/plants12173168

**Published:** 2023-09-04

**Authors:** Steven Kuan-Hua Huang, Paolo Robert P. Bueno, Patrick Jay B. Garcia, Mon-Juan Lee, Kathlia A. De Castro-Cruz, Rhoda B. Leron, Po-Wei Tsai

**Affiliations:** 1 Department of Medical Science Industries, College of Health Sciences, Chang Jung Christian University, Tainan 711, Taiwan; 7224837@mail.cjcu.edu.tw (S.K.-H.H.); mjlee@mail.cjcu.edu.tw (M.-J.L.); 2 Division of Urology, Department of Surgery, Chi Mei Medical Center, Tainan 711, Taiwan; 3 School of Medicine, College of Medicine, Kaohsiung Medical University, Kaohsiung 807, Taiwan; 4 Department of Biochemistry and Molecular Biology, College of Medicine, University of the Philippines Manila, Metro Manila 1000, Philippines; pr.bueno@themanilatimescollege.com; 5 School of Medicine, The Manila Times College of Subic, Zambales 2222, Philippines; 6 Department of Chemistry, College of Science, Adamson University, Metro Manila 1000, Philippines; 7 School of Chemical, Biological, and Materials Engineering and Sciences, Mapúa University, Metro Manila 1002, Philippines; pjbgarcia@mymail.mapua.edu.ph (P.J.B.G.); kadecastro@mapua.edu.ph (K.A.D.C.-C.); rbleron@mapua.edu.ph (R.B.L.); 8 School of Graduate Studies, Mapúa University, Metro Manila 1002, Philippines

**Keywords:** *Osmanthus fragrans* (Thunb.) Lour., prostate cancer, phytochemicals, NO scavenger, network pharmacology

## Abstract

*Osmanthus fragrans* (Thunb.) Lour. flowers (OF-F) have been traditionally consumed as a functional food and utilized as folk medicine. This study evaluated the antioxidant, anti-inflammatory and cytotoxic effects of OF-F extracts on prostate cancer cells (DU-145) and determined possible protein-ligand interactions of its compounds in silico. The crude OF-F extracts—water (W) and ethanol (E) were tested for phytochemical screening, antioxidant, anti-inflammatory, and anti-cancer. Network and molecular docking analyses of chemical markers were executed to establish their application for anticancer drug development. OF-F-E possessed higher total polyphenols (233.360 ± 3.613 g/kg) and tannin (93.350 ± 1.003 g/kg) contents than OF-F-W. In addition, OF-F-E extract demonstrated effective DPPH scavenging activity (IC_50_ = 0.173 ± 0.004 kg/L) and contained a high FRAP value (830.620 ± 6.843 g Trolox/kg). In cell culture experiments, OF-F-E significantly reduced NO levels and inhibited cell proliferation of RAW-264.7 and DU-145 cell lines, respectively. Network analysis revealed *O. fragrans* (Thunb.) Lour. metabolites could affect thirteen molecular functions and thirteen biological processes in four cellular components. These metabolites inhibited key proteins of DU-145 prostate cancer using molecular docking with rutin owning the highest binding affinity with PIKR31 and AR. Hence, this study offered a new rationale for *O. fragrans* (Thunb.) Lour. metabolites as a medicinal herb for anticancer drug development.

## 1. Introduction

Prostate cancer (PCa) is the most common non-skin cancer type and the second leading cause of cancer-related mortality in men [1,2]. This disease is characterized by uncontrolled prostate cell division, which results in aberrant cell differentiation and metastasis [1]. Other factors such as inflammation and oxidation have also been linked to cancer pathogenesis [3]. According to the National Cancer Institute, the incidence of prostate cancer has been increasing worldwide with estimated new cases of nearly 200,000 individuals, and about 30,000 deaths from the disease in 2019 [4].

The standard treatment for localized prostate cancer is the removal of the prostate through surgery and or radiotherapy. Whereas metastatic prostate cancer involves androgen deprivation therapy (ADT). However, tumor cells adapt and progress to highly aggressive forms by developing resistant mechanisms allowing them to continue proliferation [5]. Advanced and metastatic cancers have no existing curative options and are limited to hormone ablation techniques and palliative care [6]. Moreover, current methods have been reported with different adverse reactions [1]. Thus, safer alternative treatments are necessary for efficient PCa treatment.

Herbal medicines have been shown to hold immense biological and pharmacologic potentials (e.g., antioxidant, anti-inflammatory, anticancer, and immunomodulatory characteristics), which gained interest throughout the years [7,8,9]. The traditional herb, *Osmanthus fragrans* (Thunb.) Lour., is considered one of the top ten notable flowers in China, documented in Compendium of Materia Medica as early as the Ming Dynasty (AD 1368-1644) [7]. Its flower has been used as food additives and as folkloric medicine for asthma, cough, toothache, and diarrhea [8]. It was also reported to contain anti-cancer effects [9,10]. However, there is limited information about the flower’s potential bioactivities. Thus, the present study evaluated the chemical profile, antioxidant, anti-inflammatory and anticancer activities of polar (ethanol and water) extracts of *O. fragrans* (Thunb.) Lour. In addition, through the utilization of marker substances found in *O. fragrans* (Thunb.) Lour. flower extracts, we explored the possible interactions of these metabolites on PCa using in silico network pharmacology and molecular docking techniques.

## 2. Results

### 2.1. Total Phytochemical Analysis

Ethanol extract of *O. fragrans* (Thunb.) Lour. was observed to obtain higher amounts of polyphenols and tannin contents than water extract (Table 1). Whereas relatively similar flavonoid concentrations (*p* > 0.05) were found in both crude extracts.

### 2.2. Antioxidant Activity

Crude polar extracts of *O. fragrans* (Thunb.) Lour. was subjected to 2,2-diphenyl-1-picrylhydrazyl (DPPH) assay free radical scavenging and ferric reducing antioxidant power (FRAP) assays. The ethanol extracts (OF-F-E) of *O. fragrans* (Thunb.) Lour. flowers exhibited higher radical scavenging activity than water (OF-F-W) extracts as seen in Table 2. Moreover, its activity is comparable to the ascorbic acid standard (*p* > 0.05). In addition, OF-F-E showed almost twice the FRAP value of OF-F-W, indicating its superior reducing ability.

### 2.3. Anti-Inflammatory Activities

To determine the anti-inflammatory effects of the *O. fragrans* (Thunb.) Lour. polar extracts and nitric oxide levels were measured in lipopolysaccharide (LPS)-stimulated RAW 264.7 cells. Treatment with OF-F-E showed a significant NO level reduction (*p* < 0.05) compared to the control LPS-stimulated RAW 264.7 cells. OF-F-W, on the other hand, did not show significant NO reduction (Table 3). It was also observed that no obvious cytotoxic effects were found after 24 h treatment of both OF-F-W and OF-F-E.

### 2.4. Anti-Prostate Cancer Activity

**Figure 1:**
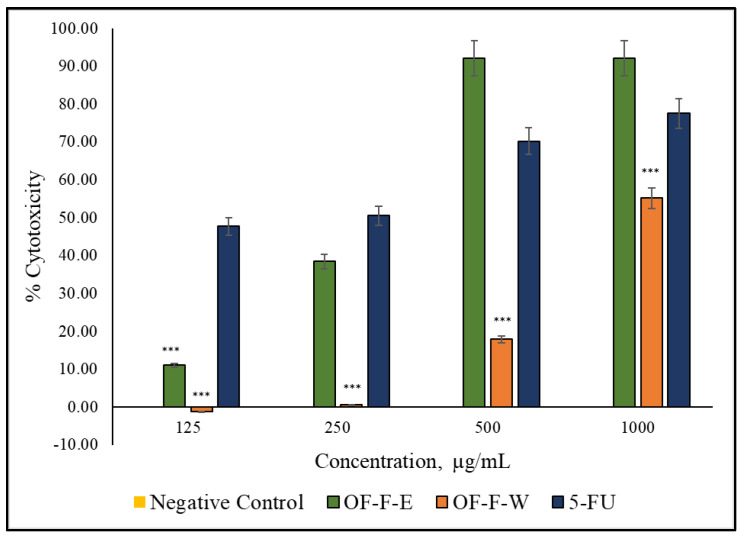
Cytotoxicity of *O. fragrans* (Thunb.) Lour. crude extracts. Values mean ± standard error of the mean (*n* = 3). Media with 0.5% DMSO and 5–flurouracil (5–FU) were used as negative and positive controls, respectively. Comparisons with 5–FU (***) *p* < 0.001.

Dose-response behavior was observed for both samples with OF-F-E exhibiting significant inhibitions than the negative control (*p* < 0.05). In Figure 1, the 500 µg/mL dose of OF-F-E has higher cytotoxicity than that of 5–fluorouracil (5–FU). It can be observed that higher half-maximal cytotoxic concentrations (IC_50_) were observed in OF-F-E (IC_50_ = 0.261 mg/mL) than that in OF-F-W (IC_50_ = 1.684 mg/mL). From this, it can be inferred that the cytotoxic potential of O. fragrans to DU-145 cells is found in the ethanol extract.

### 2.5. Network Pharmacology

**Figure 2:**
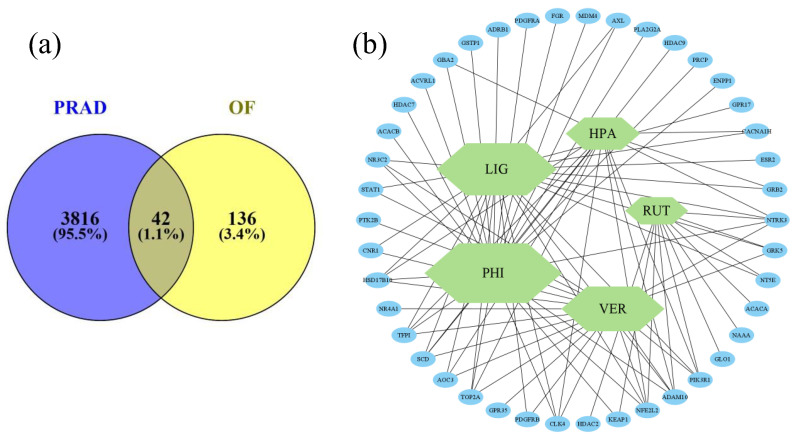
(**a**) Gene set relationship between PRAD and selected *O. fragrans* (Thunb.) Lour. metabolites; (**b**) compound-target network, where PHI = phillygenin, LIG = ligustroside, VER = verbascoside, HPA = 4-hydroxyphenyl acetate, and RUT = rutin.

Five major metabolites of OF-F-E were used. A total of 178 predicted gene targets of these metabolites were determined after duplicate removal. Gene intersections between PRAD and *O. fragrans* (Thunb.) Lour. metabolites (Figure 2a) resulted in 42 gene targets potentially associated with DU-145 cytotoxicity. In this constructed network analysis (Figure 2b), a larger hexagon corresponds to a higher degree of compound-target interaction from identified gene intersections. Of which, phillygenin and ligustroside may have the most targeted effect on the anti-prostate cancer activity of *O. fragrans* (Thunb.) Lour.

**Figure 3:**
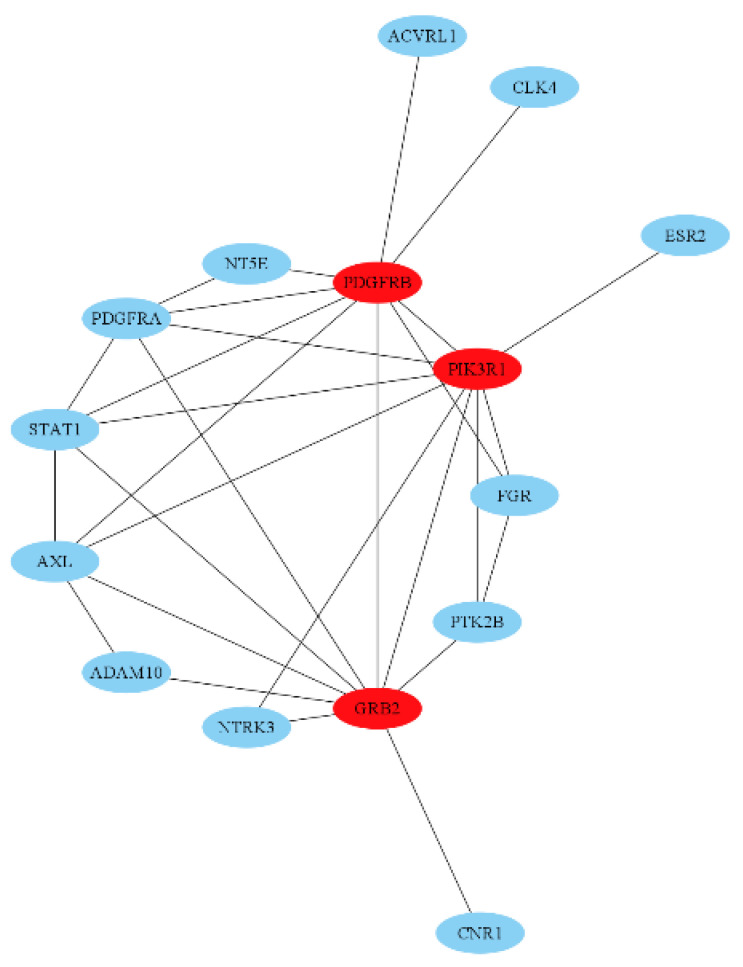
Protein-protein interaction network (PIN) of identified proteins. PIN of intersected genes set with interaction score at least 0.90 where red labelled proteins are top-ranking in interactivity.

The interaction of proteins from protein-coding genes within the identified gene intersections was constructed with degree ranking as shown in Figure 3. The top three (3) targets from topological analysis in Cytoscape were considered important targets for prostate cancer inhibition. These targets include phosphoinositide-3-kinase regulatory subunit 1 (PIK3R1), Growth Factor Receptor Bound Protein 2 (Grb2), and Platelet-derived growth factor receptor beta (PDGFRB). In addition to these targets, androgen receptor (AR) was also included for further simulations due to its important role in prostate cancer cell growth. These targets were therefore utilized for molecular simulations.

**Figure 4:**
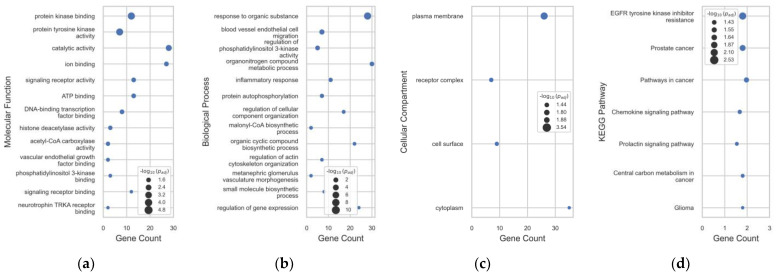
(**a**–**c**) Gene ontology term and (**d**) KEGG pathway enrichment analysis of OFF metabolite targets to PRAD.

The gene ontology (GO) terms classify expressed properties from genes and their products mainly at the first level according to their molecular functions (MF), involvement in biological processes (BP), and cellular compartmentalization (CC). There were 13 MFs, 13 BPs, and 4 CCs considered significant GO terms from 42 intersecting gene targets (Figure 4). Biological processes significantly involved may be the effect of OF-F metabolites on blood vessel endothelial cell migrations, responses to organic substances, and regulation of PI3K mainly distributed in the plasma membrane and receptor complexes. These results reveal that the OF-F metabolites may inhibit the activity of protein kinase binding and tyrosine kinase activity.

### 2.6. Molecular Docking Analysis

**Figure 5:**
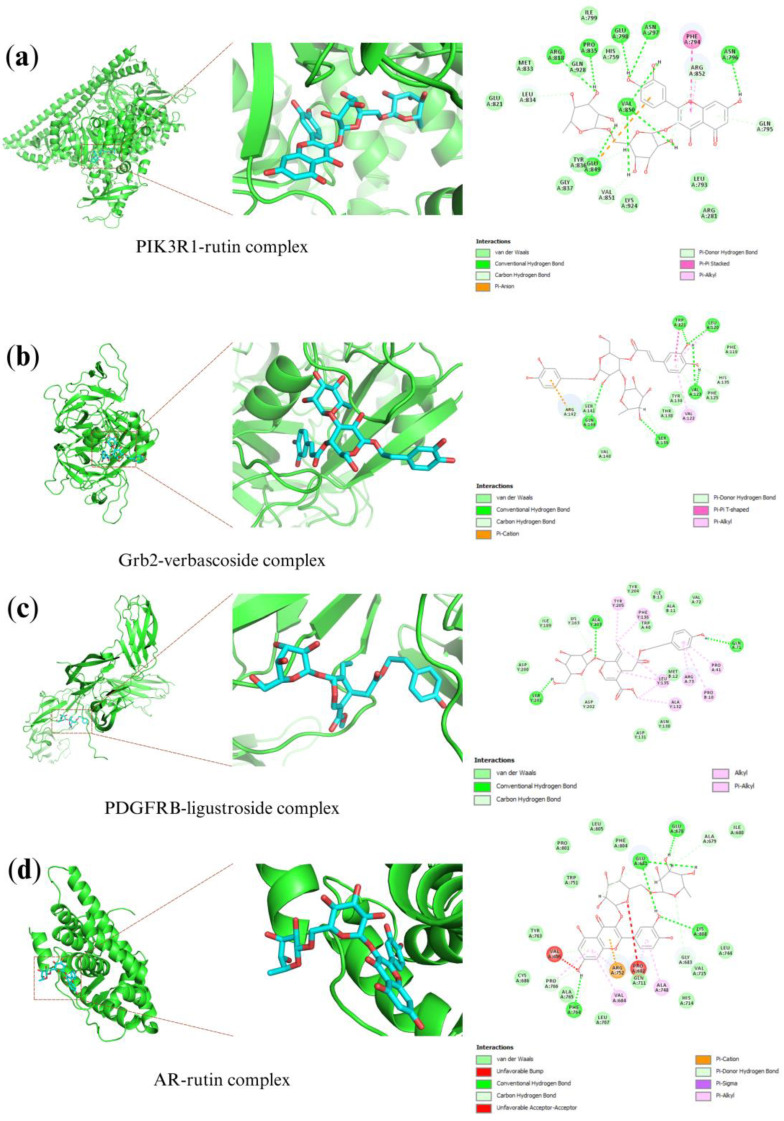
Molecular docking of OFF major metabolites and compound-target interactions. The protein-ligand complexes are (**a**) PIK3R1-rutin, (**b**) Grb2-verbascoside, (**c**) PDGFRB-ligustroside, and (**d**) AR-rutin.

Compound-target interactions were evaluated based on the LibDock score, where higher values correspond to a greater probability of ligand binding to the target protein related to prostate cancer. Five (5) selected O. fragrans metabolites and four (4) protein targets were considered in the molecular simulation, as shown in Table 4. The PIK3R1-rutin complex (PDB code: 4JPS) binds with 15 hydrogen bonds at amino acid residues GLN795, ASN796, ASN797, GLU798, ARG818, LEU834, PRO835, GLU849, VAL850, VAL851, and ARG852 with a LibDock score 2.63 times higher than the PIK3R1-5–FU complex. Whereas the Grb2-verbascoside complex (PDB code: 1GRI) with LibDock score of 115.42. For the PDGFRB-ligustroside complex (PDB code: 3MJG) with a LibDock score 2.27 times higher than the control. Lastly, the AR-rutin complex (PDB code: 5T8E) with a LibDock score 2.59 times higher than the control. These compound-target interactions revealed good binding affinity with the proteins (Figure 5), which verified protein-ligand interaction in the network.

## 3. Discussion

In recent years, there has been a growing interest in the exploration of bioactive metabolites from plant foods due to their beneficial effects in alleviating the pathogenesis of diverse human diseases ranging from cancer [10], microbial infection [11], cardiovascular ailments [12], and metabolic disorders [13]. Plant-based foods are good sources of secondary metabolites with various characteristics and properties, presenting them as important molecules in the field of medicine [14].

One of the major families of secondary metabolites known to exhibit different biological activity is phenolic compounds. Phenolic compounds are a large family of hydroxyl-containing aromatic organic compounds bearing at least one benzene ring specifically flavonoids, phenolic acids, tannins, coumarins, and lignans [15]. The number of hydroxyl groups attached to the aromatic ring directs the antioxidative capacities of phenolics [16]. In fact, phenolics exhibit relevant physiological activities including anti-inflammation [15,17] and inhibition of regulatory and metabolic enzymes associated with the development of human diseases [15,17]. Under this large group are flavonoids, which contain the basic diphenylpropane (C6-C3-C6 skeleton) and include several subgroups, such as flavones, isoflavones, flavonols, flavanones, catechins, and anthocyanin among others [15]. Flavonoid-rich plant food has been associated with several benefits to human health such as anti-inflammatory [18], antimicrobial [19], antiviral [20], anticancer and immunomodulatory activities [21]. Some flavonoids could polymerize into larger molecules such as condensed tannins (CT). CT has been reported to act as increased survivability against hypoxic stress [22]. Sharifi-Rad et al. [23] also pointed out that tannins possess anti-inflammatory and antiproliferative properties. Briefly for other phenolics, diverse phenolic acids were attributed to have, but not limited to, anti-inflammatory, anticancer, and antioxidant activities [24]. For coumarins, they are attributed to have apoptosis-inducing activity and as a multidrug resistance reversal agent [25]. Lastly, lignans were observed to reduce metastasis and proliferation of cancer cells, and reportedly have antioxidative, anticancer, anti-inflammatory, antimicrobial, and immunosuppressive activity [26].

The pharmacologic activities of polyphenols may be attributed to their specific structural component. The presence of ortho- or para-hydroxyl substituents [16], as well as the highly conjugated system and certain hydroxylation patterns [27] (e.g., a 3-hydroxy group in flavonols) are efficient electroactive potentials responsible for eliminating various free radical systems. Moreover, the extent of bioactivity of phytoconstituents is greatly affected by the choice of solvent during extraction [28]. Comparing the two crude extracts, OF-F-E contained higher polyphenols and tannin contents than OF-F-W. This result agreed with previous studies of Galgano et al. [29] and Hussain et al. [30], where the highest number of polyphenolic compounds were attained using ethanol as extracting solvent for lentil seed coats and *Adansonia digitata* fruit pulp, respectively. Ansori et al. [31] found a 10-fold tannin yield increase from *Medinella speciosa* ethanol fruit extract. Ethanol penetrates the plant tissue matrix better than water, which allows maximal extraction of secondary metabolites [32]. Meanwhile, relatively similar flavonoid concentrations, may be attributed to the solubility of carbohydrate-containing secondary metabolites such as flavonoid glycoside derivatives extracted with more polar solvents, such as water [30]. Since these extraction solvents solubilize compounds with varying polarities, their pharmacokinetics and pharmacodynamics are potentially widely different. Since ethanol extracts dissolve lipophilic compounds, their antioxidant properties are inclined to protect against lipid oxidation. Whereas water extracts with potent antioxidant properties could scavenge free radicals and reduce ROS that are water-soluble. However, for cancer treatment and prevention, it is important to cross the lipid bilayer of the cancer cell membrane since common targets for this therapeutic effect are intracellular.

Plants hold a wide array of natural compounds that combat reactive oxygen species (ROS). When ROS is in excess in the body, this may progress to degradative oxidative reactions and gene and biomolecule structural modification, resulting in oxidative stress-induced diseases such as cancer [33]. To balance the excess generated ROS, exogenous antioxidants are incorporated into the diet, which terminates radical chain reactions [34]. However, increased oxidative stress causing an imbalance of ROS and antioxidants promotes carcinogenic mechanisms and genetic mutations which could potentially trigger oncogenic signaling pathways [35,36]. In this study, higher antioxidative activity of OF-F-E may then be attributed to the greater abundance of polyphenols, and condensed tannins in the extract. Hung et al. [37] determined that *O. fragrans* ethanol extract showed significant DPPH inhibition (IC_50_ = 8.4 μg/mL) with a significant amount of total phenolic contents (367.9 ± 13.4 µg/mL). This was confirmed by Wang et al. [8] highlighting a strong positive relationship between phenol components in *O. fragrans* flowers and the antioxidant potential with a correlation coefficient >0.80. Studies reported that high levels of polyphenols are correlated with high antioxidant properties. This is due to the presence of double bonds within the aromatic ring and hydroxyl groups attached to the structure [38]. These structures enable polyphenols to decrease the rate of oxidation through deactivation of active radicals and their precursors. Commonly, they neutralize free radicals by donating an electron to impede chain reactions [34]. Conversely, the influence of flavonoids on its antioxidative activity may be minimal as OF-F-W and OF-F-E showed relatively similar amounts of TF. Hence, these results revealed the OF-F-E contained promising antioxidant compounds that may contribute to other pharmacological activities.

Nitric oxide (NO) is a highly reactive free radical mediating increasing edema and prostaglandin elevation during the late phase of inflammation [39]. To address this inflammatory cascade, extracts can act as NO scavengers to inhibit NO production. Treatment with OF-F-E showed a significant NO level reduction. On the other hand, OF-F-W exhibited no NO reduction in LPS-stimulated RAW cells. It was also observed that both OF-F-W and OF-F-E have no obvious cytotoxic effects on RAW 264.7 cells after 24 h, even at the highest concentration. This finding is similar to the results reported by Lee et al. [40] where *O. fragrans* var. *auruantiacus* flowers showed strong inhibitory activity against NO production in LPS-stimulated RAW 264.7 cells. In addition, inhibition was associated with isolated compounds such as phillygenin and phillyrin [40].

Inflammation and oxidation have been linked as key factors in cancer pathogenesis [3]. During PCa development, low oxygen conditions (hypoxia) in prostate cells have been reported [41]. Under this hypoxic environment, tumor cells secrete high levels of inflammatory chemokines [42]. These tumor-derived molecules induce the accumulation and differentiation of tumor-associated macrophages, which produce ROS and RNS in the tumor cells [43,44]. This promotes a repetitive sequence of inflammation-oxidation processes in cancer [43,44]. Hence, inhibition of inflammation and termination of oxidative processes may be a valid method for impeding the progression of PCa. In the present study, ethanolic extract of *O. fragrans* (Thunb.) Lour. flowers have a higher cell cytotoxic activity in a concentration-dependent manner in contrast to 5–fluorouracil (5–FU). Similar cytotoxic activity of *O. fragrans* var. *auruantiacus* flowers was reported against HCT-116 human colon cancer cells [45]. Verbascoside, an active compound from *O. fragrans*, has shown the treatment of human prostate cancer cell lines (PC-3 and DU-145) by suppressing cell proliferation, tumor invasion, and epithelial-to-mesenchymal transition [9].

Genomic alterations and mutations have been the foundation of carcinogenesis resulting in either oncogenes with gain of function or tumor suppressor genes having recessive loss of function [46]. In addition, research in the past decades has shown that continuous exposure to oxidative stress can lead to chronic inflammation and can initiate the onset of chronic diseases such as cancer [3,47]. Oxidative stress can activate transcription factors leading to the expression of genes such as those involved in the production of growth factors, inflammatory cytokines, cell cycle regulation molecules, chemokines, and anti-inflammatory mediators [47]. Given these complex systems that take place in cancer, the use of single targeted drugs only resolves one aspect of the disease hence, a highly effective drug should target multiple facets of the disease while being selective and sparing normal cells in tissues and/or organs [48,49,50]. The results of this study have shown the polypharmacological potential of OF-F-E. Hence, a network pharmacology and molecular docking approach was executed to further understand the effects of OF-F-E on PCa.

Network pharmacology is an emerging discipline oriented towards a multi-goal-multi-disease paradigm which aims to understand drug action and interaction between multiple targets, such as G-protein coupled receptors [48], key regulatory enzymes [49], nuclear receptors, and transcription factors [51]. It makes use of computational power to systematically evaluate and understand compound-gene and/or compound-protein interactions in a living cell. It can also help to understand and explore the synergistic effects of bioactive compounds in disease and its multiple pathways [52,53]. In the study, five major metabolites were used in the subsequent bioinformatics analysis. These five metabolites were reported as the major phytochemical constituents of *O. fragrans* methanolic flower extract [54]. Albeit different, the polarities of methanol and ethanol are close to each other; thus, these metabolites were chosen. The results of the study showed that the metabolites chosen have an interaction with three protein coding genes which include PIK3R1, Grb2, and PDGFRB.

The phosphatidylinositol 3-kinase (PI3K) pathway plays an important signaling pathway in cancer which is responsible for regulation of cell growth, metabolism, survival, proliferation, and angiogenesis [55]. The PIK3R1 gene is responsible for encoding the regulatory subunit of the PI3K enzyme and plays an important role in the regulation of the enzyme’s catalytic activity [56]. In cancer, changes in the PIK3R1 gene result in alteration of the regulatory subunit of the enzyme which results in loss of control in the enzyme’s activity [56,57]. In a study by Chakraborty et al. [58], alterations in the PIK3R1 in prostate cancer led to a high PI3K-AKT metabolic activity while reduced mRNA expression was observed in advanced stages of prostate cancer.

Another protein coding gene that showed significant interaction with the OF-F-E was Grb2. Growth Factor Receptor Bound Protein 2 (Grb2) is an adapter protein that functions in cell cycle progression, actin-based cell motility, angiogenesis, vasculogenesis, and epithelial morphogenesis [59]. It also acts as an important downstream intermediary in some tyrosine-kinase-dependent and oncogenic signaling pathways [60,61]. This protein has a structure that includes one Src homology 2 (SH2) domain flanked by two SH3 domains [62]. The former interacts with receptor tyrosine kinases (i.e., platelet-derived growth factor receptor), non-receptor tyrosine kinases (i.e., focal adhesion kinase (i.e., FAK, Bcr/Abl), and some substrates of tyrosine kinases while the latter binds with proline-rich regions of the interacting protein [59]. In cancer, it is involved in signal transduction of oncogenic tyrosine kinases which are attributed to the development and progression of the disease [63]. Studies have reported that increased expression and overexpression of Grb2 relate to poor prognosis in patients with gastric cancer and lymph node metastasis and poor survival in esophageal cancer patients, respectively [64,65]. It was also hypothesized that overexpression of Grb2 could be used as a novel poor prognostic biomarker in patients with prostate cancer [63].

The involvement of platelet-derived growth factor ligands (PDGFs) in PCa was also emphasized in the results. PDGFs and their receptors (PDGFRs) play a central role in the regulation of cell growth and division [66]. They are considered strong mitogens of cells having mesenchymal origin [67] and are involved in angiogenesis [68]. The binding of PDGFs to PDGFRs results in cellular autophosphorylation [69], followed by activation of signal transduction molecules such as Grb2 [68] and PI3K [70,71], leading to PCa progression. A study also showed that expression of PDGFR-β was linked with a greater prostate-specific antigen failure rate to those diagnosed with localized PCa and treated by radical prostatectomy [72].

This study involving network pharmacology was coupled with molecular docking studies to investigate ligand-receptor binding interaction and assess its binding affinity and stability [73]. Among the metabolites, rutin has shown strong affinity with PIK3R1 and AR. Rutin is a bioflavonoid that can be found in vegetables, fruits, medicinal herbs, and plant-based beverages [74]. In vitro studies have shown its anti-cancer potential against a wide variety of human cancers such as those found in the colon [75,76,77], lungs [75,76,77], prostate, cervical, pancreas and others [75,76,77]. This phytochemical counteracts cancer through several mechanisms such as induction of apoptosis, cell cycle arrest, modulation of angiogenesis, regulation of inflammation and oxidative stress, and inhibition of malignant cell growth [74]. These mechanisms are usually attributed to the regulation of cell signaling pathways [74]. ARs are considered to have the biggest contribution to PCa pathology [78,79]. PCa development and progression are generally dependent on androgenic stimulation [78,79]. With such, treatment modalities for PCa are centered on depriving tumors of androgens or blocking their actions [78,79]. However, this treatment could be temporary, and patients could eventually relapse and develop castration-resistant PCa as a result of overexpression and/or mutations in the ARs [79]. Studies have shown that rutin can be used as a potential treatment for androgen-sensitive PCa because its mechanism of action revolves around the suppression of androgen biosynthesis and metabolism [80].

On the other hand, verbascoside was also shown to have a strong affinity for Grb2. A few studies have shown that verbascoside has strong anti-proliferative activity [81]. It was able to promote rat prostate apoptosis and prevent the development of benign prostatic hyperplasia [82]. It was also able to inhibit cell proliferation and migration of prostate tumor cell lines, PC3 and DU-145, by suppressing the TGF-β signaling pathway and as well as the epithelial-mesenchymal transition (EMT) changes brought upon by the HMGB1/RAGE axis [9]. Lastly, strong binding between ligustroside and PDGFR-β was reported. Studies on the effect of ligustroside in cancer are scarce. Phenolic compounds found in olive oil, where ligustroside is a component, have angiopreventive potential against urologic cancers [83].

## 4. Materials and Methods

### 4.1. Plant Collection and Preparation of Plant Extracts

The flowers of *Osmanthus fragrans* were acquired from a traditional Chinese medicine store in Tainan City, Taiwan. Voucher specimens were deposited as #CJCU-OFF-001, at the Department of Medical Science Industries at Chang Jung Christian University, Taiwan.

The flowers of *O. fragrans* were mechanically crushed using a blender. The plant samples were subjected to two-hour extraction using 95% ethanol in a 1:20 ratio under reflux at 333.15 K. For the water extract, samples were allowed to boil in a traditional Chinese decoction pot until the volume of the water was reduced to approximately 200 mL. The extracts were filtered by vacuum filtration and were concentrated using a rotary evaporator. After which, crude extracts were placed in a freeze-dryer until the solvent was completely liberated. The samples were then stored in at 253.15 K refrigerator prior to use for further analysis.

### 4.2. Total Phytochemical Analysis

#### 4.2.1. Total Phenolic Content (TPC) Assay [16]

A gallic acid (GA) stock solution (1000 µg/mL) was prepared by dissolving five milligrams (5.0 mg) of gallic acid standard in five milliliters of ethanol. The stock solution was subjected to two-fold dilution to obtain working standard solutions (31.25–500 µg/mL). In a 96-well microplate, twenty microliters (20 µL) of sample/standard solutions were sequentially treated with 100 µL Folin’s reagent and 80 µL Na_2_CO_3_ and left in the dark for 30 min. The absorbances were measured at 600 nm using an ELISA microplate reader. Each test solution was prepared in triplicates.

#### 4.2.2. Total Flavonoids Content (TFC) Assay [16]

A rutin stock solution (1000 µg/mL) was prepared by dissolving five milligrams (5.0 mg) of rutin standard in five milliliters of ethanol. The stock solution was subjected to two-fold dilution to obtain working standard solutions (31.25–500 µg/mL). In a 96-well microplate, the sample/standard solutions (100 μL) reacted with 100 μL of 2.0% (*w*/*v*) AlCl_3_ reagent and were incubated for 15 min at room temperature. The absorbances were measured at 430 nm using an ELISA microplate reader. Each test solution was prepared in triplicates.

#### 4.2.3. Total Tannin Content (TTC) Assay [16]

A catechin stock solution (1000 µg/mL) in ethanol was prepared by dissolving five milligrams (5 mg) of catechin standard in ethanol. The stock solution was subjected to two-fold dilution to obtain working standard solutions (31.25–500 µg/mL). Three hundred microliters (300 µL) of sample/standard solutions were placed in a clean test tube. To all reaction mixtures, 600 μL of 1% (*w*/*v*) vanillin in 80% (*v*/*v*) H_2_SO_4_ reagent. The reaction mixture was allowed to stand for 15 min. An aliquot of 200 µL was transferred into a 96-well microplate. The absorbances were measured at 530 nm using an ELISA microplate reader. Each test solution was prepared in triplicates.

### 4.3. Antioxidant Activity

#### 4.3.1. DPPH Free Radical Scavenging Assay [16]

In a 96-well microplate, 50 µL of each test solution was added followed by 150 µL DPPH solution. The reaction mixture was allowed to stand at room temperature for 30 min in the dark. Absorbance at 517 nm was measured using an ELISA microplate reader. The radical scavenging activity (%RSA) was calculated using Equation (1) to determine the 50% inhibitory concentration (IC_50_). Ascorbic acid was used as the positive control.

(1) $$\%\;RSA=\frac{\left(A_{ctl}-A_{blk}\right)-\left(A_{spl}-A_{blk}\right)}{\left(A_{ctl}-A_{blk}\right)}\times 100$$

*%RSA*: % Radical scavenging activity.

*A_ctl_*: Absorbance of control sample.

*A_blk_*: Absorbance of blank.

*A_spl_*: Absorbance of sample.

#### 4.3.2. Ferric Reducing Antioxidant Power (FRAP) Assay [16]

Varying concentrations of Trolox standard solutions were prepared by two-fold serial dilution. Fifty microliters (50 µL) of sample/standard solutions were treated with 1450 µL of FRAP reagent in a clean microcentrifuge tube. The reaction mixture was allowed to stand at room temperature for 30 min in the dark. Absorbance at 593 nm was measured using an ELISA microplate reader. The antioxidant capacity was expressed in FRAP unit, in mg Trolox/g calculated by linear regression curve.

### 4.4. Anti-Inflammatory Activity

#### 4.4.1. Murine Macrophage Cell Line RAW 264.7 Culture

Murine macrophage cell line RAW 264.7 was obtained from the Bioresource Collection and Research Center (BCRC, Taiwan). The cells were cultured in a T-25 culture flask with Dulbecco’s modified Eagle’s medium (DMEM), containing 10% fetal bovine serum (FBS), 1% penicillin-streptomycin (Gibco, Thermo Fisher Scientific, Waltham, MA, USA), and kept at 37 °C in a humidified atmosphere containing 5% CO_2_. After reaching 70–80% confluence, the cells were sub-cultured within two-day intervals until nitric oxide tests were performed.

#### 4.4.2. Measurement of NO Production by Griess Reaction and Cell Viability [84,85]

RAW 264.7 cells were cultured in 96 well plates (4 × 10^4^ cells/well) and incubated at 37 °C. After 24-h incubation, the cells were treated with various concentrations of rattan shoot extracts/fractions in the presence of lipopolysaccharide (LPS; 1 μg/mL) for another 24 h. Cell supernatants were collected and were mixed with Griess reagent in a 100 µL reaction mixture (50 µL of cell supernatant and 50 µL of Griess reagent) for 20 min. Absorbance was measured at 540 nm using a microplate reader. The cells without treatment served as the blank control. The cells treated with LPS in the absence of the sample served as the negative control (LPS group).

Cell viability was determined using the WST-1 assay (Abcam^TM^, Cambridge, UK). Briefly, 100 µL serum, LPS-free medium was placed in each treated RAW 264.7 cells. Ten (10 µL) WST-1 reagent was added to each well. The plate was incubated for 2 h at 37 °C. The absorbances were measured at 570 nm. All experiments will be performed three times in triplicates.

### 4.5. Anti-Prostate Cancer Activity

#### 4.5.1. Human Prostate Cancer Cell Line DU-145

Human prostate cancer cell lines DU145 were obtained from Bioresource Collection and Research Center. were cultured in Eagle’s Minimum Essential Medium (EMEM), containing 10% fetal bovine serum (FBS), 1% penicillin-streptomycin, and kept at 37 °C in a humidified atmosphere containing 5% CO_2_. The cells were sub-cultured within two-day intervals after reaching 70–80% confluence.

#### 4.5.2. Cell Treatment and Cell Viability with WST-1 Assay [86]

DU-145 cells were cultured in 96-well microarray plates (2 × 10^4^ cells/well) and incubated at 37 °C. After 24-h incubation, the cells were treated with various concentrations (two-fold dilution from 1000 to 125 µg/mL) of crude extracts for another 24 h. The 5–fluorouracil (5–FU) was used as the positive control. Cell viability was determined using the WST-1 assay (Abcam^TM^). Briefly, 100 µL fresh medium was placed in each treated well with DU-145 cells. Ten (10 µL) WST-1 reagent was added to each well. The plate was incubated for 2 h at 37 °C. The absorbances were measured at 570 nm. Measurements were performed in triplicates.

### 4.6. Data Treatment and Statistical Analysis

GraphPad Prism software was used for statistical analysis and data visualization. Experimental data are reported as mean ± standard deviation (SD). Multiple comparisons of means were conducted through one-way ANOVA. A statistical significance of *p* < 0.05 was set throughout the analysis.

### 4.7. Network Pharmacology

#### 4.7.1. Target Prediction and Identification

The predicted gene targets of the major compounds identified from *O. fragrans* extracts were identified from Super-PRED (prediction.charite.de, accessed on 28 April 2023). [87]. Differentially expressed genes from prostate adenocarcinoma (PRAD) and normal prostate were identified from Gene Expression Profiling Interactive Analysis (GEPIA2, gepia2.cancer-pku.cn, accessed on 28 April 2023) and the Gene Expression Omnibus (GEO, www.ncbi.nlm.nih.gov/geo, accessed on 28 April 2023) [88,89]. Statistically significant over-expressed genes were selected from a cutoff of |log2 FC| > 1 and *p* < 0.05 using LIMMA. Gene nomenclatures were standardized into their official gene symbols by the HUGO Gene Nomenclature Committee using SynGO (www.syngoportal.org/convert, accessed on 28 April 2023) [90]. Matched gene targets between the major compounds and PRAD were selected for further analysis using Venny 2.1 (bioinfogp.cnb.csic.es/tools/venny, accessed on 28 April 2023) [91].

#### 4.7.2. Protein-Protein Interaction (PPI) Network Construction

The identified matched protein-coding genes were imported to STRING database 11.5 (string-db.org, accessed on 2 May 2023) for the construction of a protein-protein interaction network (PIN) [92]. Furthermore, proteins with more than one (1) interaction, interaction score of more than 0.4, and FDR stringency of 0.05 limited to “*Homo sapiens*” species were considered significant. The PPI network is transferred to CytoScape 3.9.1 for further analysis [93]. Protein relevance in the network was evaluated with topological analysis using degree ranking from the cytoHubba plug-in in CytoScape [94].

#### 4.7.3. Gene Ontology Term and KEGG Pathway Enrichment Analysis

Functional profiling was performed with g:Profiler (biit.cs.ut.ee/gprofiler/gost, accessed on 2 May 2023) for exploration of predicted pharmacological mechanism and signaling pathways involved in the action of metabolites to the selected targets [95,96]. Relevant results of gene ontology (GO) terms, KEGG and Reactome pathways were characterized from a g:SCS significance threshold of less than 0.05 limited to “*Homo sapiens*” species. Secondary data filtering of GO terms were automatically performed from g:Profiler with a simple greedy search algorithm. 

#### 4.7.4. Molecular Docking Validation

The molecular modeling and visualization software BIOVIA Discovery Studio was employed for the docking analysis. Two-dimensional (2D) structure-data files (SDFs) of ligand molecules identified were collected from PubChem (chem.ncbi.nlm.nih.gov, accessed on 3 May 2023). The ligands were prepared with the Prepare Ligands tool in Discovery studio for charge standardization and generation of its tautomeric and ionization states. Experimentally elucidated structures of protein targets available from the Protein Data Bank (PDB, www.rcsb.org, accessed on 3 May 2023) were collected in PDB file format. The crystal structure of identified proteins was considered as targets for molecular docking. Afterward, heteroatoms and water molecules were removed, and polar hydrogens were added to the protein structure. The protein is prepared with the Prepare Protein tool in Discovery Studio which automatically repaired and protonated the protein structure. This protein is typed with CHARMm forcefield and Momany-Rone partial charge estimation method. The docking runs were performed with the LibDock algorithm in Discovery Studio. For the docking parameters, modified settings were applied (100 hotspots, 0.25 Å RMSD tolerance, High-Quality docking preference, and BEST conformation generation method). The defined binding sites were determined from the literature [79,97,98,99,100]. The docking analysis results were evaluated according to the binding energy score compared to the control from the in vitro tests, 5–FU.

### 4.8. Statistical Analysis

The results were expressed as means ± standard deviation (SD). These were analyzed using one-way analysis of variance (ANOVA) with Dunnett’s multiple comparison test using the GraphPad Prism 7.0 (GraphPad Software Inc., San Diego, CA, USA). Statistical significance was set at *p* < 0.05.

## 5. Conclusions

Extracts of *O. fragrans* (Thunb.) Lour. extract showed antioxidative, anti-inflammatory, and anti-cancer potentials. Of which, ethanol extract had better dose-response behavior than water extract attributed to higher total polyphenols and tannin contents. Bioinformatics showed that major *O. fragrans* (Thunb.) Lour. metabolites have significant interaction with protein-encoding genes PIK3R1, Grb2, and PDGFRβ, which have significant actions in various cellular signaling pathways. Molecular docking analysis of the *O. fragrans* (Thunb.) Lour. metabolites to these key targets consistently verified the protein-ligand interactions from network pharmacology results. Hence, the observed cytotoxicity in DU-145 by ethanol flower extract may be attributed to the metabolite’s interaction with PIK3R1, Grb2, and PDGFRβ and AR. Thus, further in vivo and pharmacologic studies should be executed in order to validate the potential of a single isolated compound or synergistic behavior of crude *O. fragrans* (Thunb.) Lour. ethanolic extract against PCa.

## Figures and Tables

**Table 1:** Total phytochemical content of *O. fragrans* (Thunb.) Lour. extracts.

Sample	Total Phenolic Content (g GAE per kg Sample)	Total Flavonoid Content (g RE per kg Sample)	Total Tannin Content (g CE per kg Sample)
OF-F-W	170.860 ± 2.868	17.820 ± 0.544	38.590 ± 0.854
OF-F-E	233.360 ± 3.613	17.200 ± 0.771	93.350 ± 1.003
Equation of the line:	y = 0.0038x − 0.0132 (r^2^ = 0.9998)	y = 0.0049x + 0.0287 (r^2^ = 0.9994)	y = 0.0078x − 0.0242 (r^2^ = 0.9974)

GAE, gallic acid equivalents; RE, rutin equivalents; CE, catechin equivalents.

**Table 2:** Antioxidant activities of *O. fragrans* (Thunb.) Lour extracts.

Extract	DPPH IC_50_ (kg/L)	FRAP (Trolox g/kg)
OF-F-E	0.337 ± 0.008	467.300 ± 2.784
OF-F-W	0.173 ± 0.004	830.600 ± 6.843
Ascorbic acid	0.111 ± 0.003	-
		Trolox calibration curve: y = 0.0038x + 0.0074 R^2^ = 0.998

**Table 3:** Anti-inflammatory activities of *O. fragrans* (Thunb.) Lour extracts.

Extract	IC_30_ (mg/mL)	Cell Viability
OF-F-E	N.D.	Non toxic
OF-F-W	0.3218	Non toxic

N.D., not detected.

**Table 4:** Molecular docking of common targets between *O. fragrans* (Thunb.) Lour. metabolites and PRAD.

Protein Target	Metabolite	Number of Interactions				LibDock Score
		vdW	H-Bond	Other	Unfavorable	
PIK3R1	PHY	14	3	7	-	121.49
	LIG	17	3	9	-	136.26
	VER	14	7	4	2	162.70
	HPA	9	1	2	-	77.54
	RUT	10	15	3	-	163.22
	5–FU	6	3	1	2	63.15
Grb2	PHY	5	9	3	3	84.84
	LIG	8	3	6	-	98.15
	VER	6	10	4	-	115.42
	HPA	5	4	2	1	70.23
	RUT	6	7	8	-	104.35
	5–FU	-	-	-	-	None
PDGFRB	PHY	13	9	5	-	81.56
	LIG	9	5	9	-	136.46
	VER	12	4	5	-	129.82
	HPA	3	4	3	-	79.33
	RUT	9	5	8	1	115.99
	5–FU	4	4	4	-	60.04
AR	PHY	12	2	9	-	127.89
	LIG	10	7	11	1	165.10
	VER	7	11	7	-	169.33
	HPA	7	3	3	-	73.25
	RUT	13	12	6	2	173.17
	5–FU	5	4	2	-	66.76

## Data Availability

The data and results presented in this study are available on request from the first author and corresponding author.
